# Paradoxical Reaction With Intercostal Lymphadenopathy in Tuberculous Pleurisy

**DOI:** 10.7759/cureus.80931

**Published:** 2025-03-21

**Authors:** Manami Ichikawa, Yusuke Ito

**Affiliations:** 1 Department of General Medicine, Tokatsu Hospital, Nagareyama, JPN; 2 Department of Family Practice, Azusawa Hospital, Itabashi, JPN

**Keywords:** antitubercular agents, lymphadenopathy, paradoxical reaction, tb – tuberculosis, tuberculous pleurisy

## Abstract

Paradoxical reaction (PR) is defined as the clinical or imaging worsening of tuberculosis lesions or the emergence of new lesions after anti-tuberculosis therapy. Although additional treatments, including corticosteroids, may be considered in severe cases, the mainstay management of a PR is close monitoring, as the condition is generally self-limited. Radiographic presentations are diverse, and currently, no specific radiographic features of this condition have been identified, which may lead to misdiagnosis as treatment failure, drug-resistant tuberculosis, or another infection. PRs in tuberculous pleurisy, once considered rare, typically manifest as increased pleural effusion or progression of infiltration. We present a rare case of tuberculous pleurisy in which intercostal lymphadenopathy enlargement occurred as a PR on the 22nd day of anti-tuberculosis therapy. The symptoms did not significantly affect the patient's quality of life, physical activity, or respiratory function, and the patient was closely monitored without modifying treatment or adding corticosteroids, showing improvement on the 34th day of anti-tuberculosis therapy. Two years have passed after the completion of treatment, and no recurrence of tuberculosis or PR has been observed. This case highlights the importance of clinician vigilance for PRs in tuberculosis management. During treatment for tuberculous pleurisy, unexplained intercostal lymphadenopathy should raise suspicion of a PR, and close monitoring without invasive tests or treatments is recommended.

## Introduction

Paradoxical reaction (PR) is defined as the clinical and imaging deterioration of pre-existing tuberculosis lesions or the appearance of new lesions after initiating appropriate anti-tuberculosis therapy [1,2]. PR generally occurs 3-12 weeks after the initiation of anti-tuberculosis therapy, with various presentations including lymphadenopathy, pulmonary infiltration, and the development of pleural effusion [2-4]. The pathogenesis is thought to involve interactions between the host immune response and microbial products, including delayed hypersensitivity, reduced immune suppression, and a reaction to tuberculoprotein release triggered by anti-tuberculosis therapy [1,2]. This enhanced focal immune response recruits lymphocytes and macrophages to the lesion site, potentially contributing to pleural effusion worsening, pulmonary infiltrates, and lymphadenopathy in PR [4]. Tuberculous pleurisy is the second most common presentation of tuberculosis and typically presents with pleuritic pain, cough, and B symptoms (fever, weight loss, and night sweats) [5]. PR has historically been considered rare in tuberculous pleurisy [6], and clinical deterioration due to PR can be challenging for clinicians [7]. In this condition, PR typically manifests as increased pleural effusion or pulmonary infiltrates and may be misdiagnosed as treatment failure or a secondary bacterial infection, underscoring the need for recognition [2-4]. We present a rare case of PR with intercostal lymphadenopathy in a non-human immunodeficiency virus (HIV) tuberculous pleurisy patient.

## Case presentation

**Figure 1:**
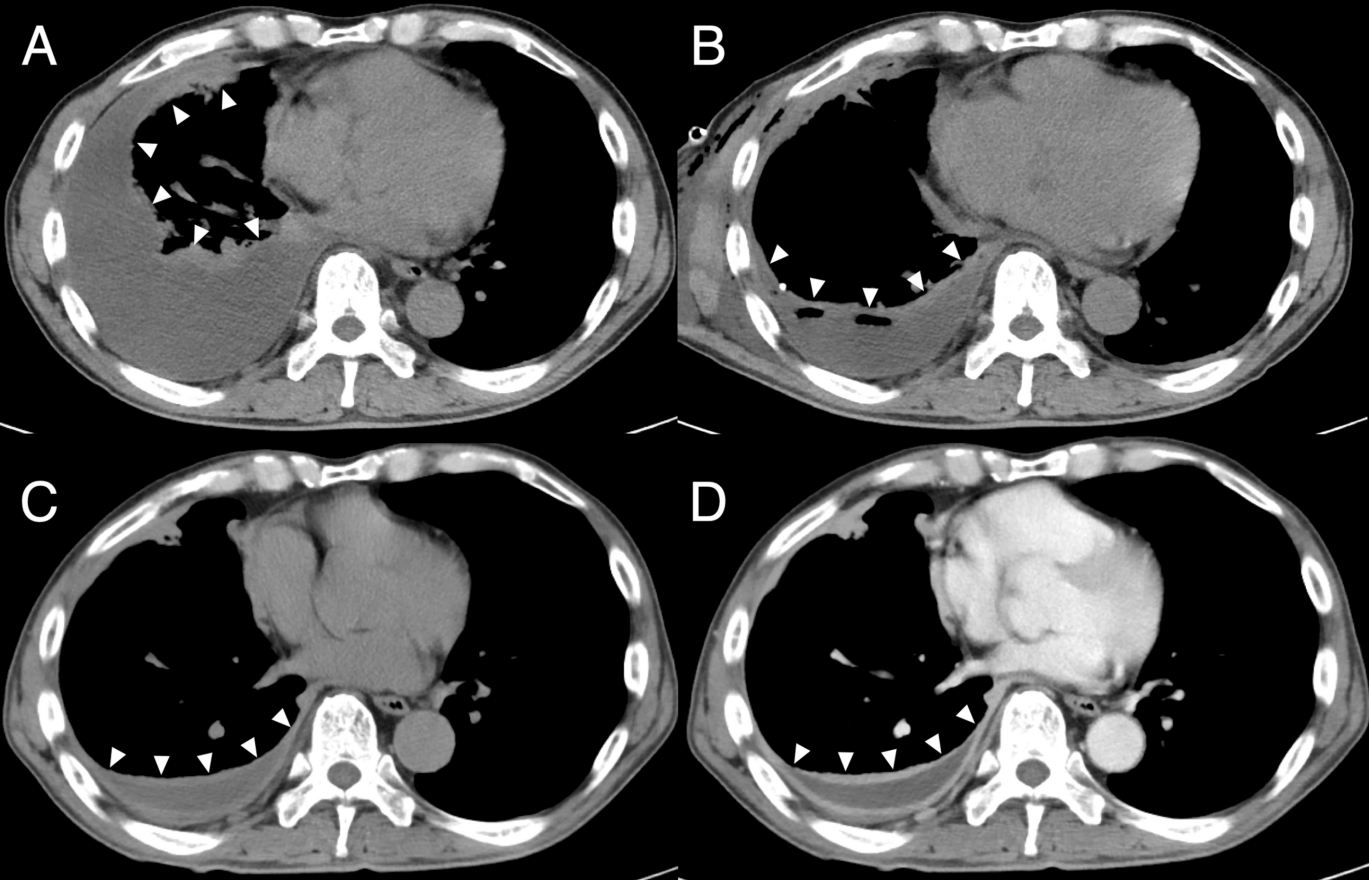
(A) CT scan on admission (seven days before the initiation of anti-tuberculosis therapy) showing right pleural effusion (white arrowheads). (B) CT scan on the second day of admission (five days before the initiation of anti-tuberculosis therapy) after pleural drainage, showing a reduction in effusion volume (white arrowheads). (C, D) CT scan on the 34th day of admission (27 days after the initiation of anti-tuberculosis therapy) (C: non-contrast, D: contrast) showing further improvement in right pleural effusion (white arrowheads).

A 69-year-old man with a family history of pulmonary tuberculosis presented with a one-week history of remittent fever and right chest pain. Upon admission, he was alert and oriented with vital signs: blood pressure of 119/68 mmHg, heart rate of 90 beats/min, body temperature of 37.9°C, respiratory rate of 16 breaths/min, and oxygen saturation of 94% on ambient air. Physical examination revealed diminished breath sounds in the right lung but no tenderness or skin abnormalities. Laboratory tests showed an elevated C-reactive protein (CRP) level of 13.5 mg/dL, a positive T-SPOT®.TB test, and a negative HIV antigen/antibody test (Table 1). Computed tomography (CT) demonstrated right pleural effusion without evidence of pneumonia or lymphadenopathy, including intercostal, cervical, axillary, or inguinal nodes (Figures 1A-1B). A thoracostomy tube was inserted, and approximately 1000 mL of pleural fluid was drained. Pleural fluid analysis revealed a lymphocyte-predominant exudative pleural effusion, with 82.8% lymphocytes and 11.4% neutrophils, and an adenosine deaminase level of 64.4 U/L (Table 2). *Mycobacterium tuberculosis* was subsequently isolated from the pleural fluid culture. Based on these findings, tuberculous pleurisy was diagnosed. On the seventh day of admission, treatment was initiated with isoniazid, rifampicin, ethambutol, and streptomycin, resulting in the resolution of fever and chest pain and improvement of CRP levels. Drug susceptibility testing confirmed that the isolated *M. tuberculosis* strain was susceptible to all these drugs.

**Table 1:** Blood laboratory test results on the admission. HIV: human immunodeficiency virus

Parameter	Result	Reference range
White cell count (/μL)	4150	3500-9000
Neutrophils (%)	62.8	40-70
Lymphocytes (%)	22.4	20-50
Monocytes (%)	13.3	3-11
Eosinophils (%)	1.0	0-5
Basophils (%)	0.5	0-2
Red blood cell (10^4^/μL)	358	427-570
Hemoglobin (g/dL)	11.9	13.5-17.6
Platelet count (10^4^/μL)	30.3	13-37
Total protein (g/dL)	6.9	6.7-8.3
Albumin (g/dL)	3.0	3.8-5.2
Urea nitrogen (mg/dL)	14.0	8-20
Creatinine (mg/dL)	1.09	0.6-1.1
Sodium (mEq/L)	135	135-148
Potassium (mEq/L)	4.5	3.5-5
Chloride (mEq/L)	99	98-110
Aspartate aminotransferase (U/L)	38	10-40
Alanine aminotransferase (U/L)	35	5-42
Lactate dehydrogenase (U/L)	223	124-222
Creatinine kinase (U/L)	187	40-200
C-reactive protein (mg/dL)	13.53	0-0.3
Glucose (mg/dL)	147	70-109
T-SPOT.TB	Positive	-
HIV antigen/antibody	Negative	-

**Table 2:** Results of the laboratory analysis of the pleural effusion. ADA: adenosine deaminase

Parameter	Result
Appearance	Yellow
Lactate dehydrogenase (U/L)	764
Glucose (mg/dL)	125
Total protein (g/dL)	5.0
Albumin (g/dL)	2.52
Cell count (/µL)	1677
Macrophages (%)	5.4
Neutrophils (%)	82.8
Lymphocytes (%)	11.4
Monocytes (%)	0.2
Eosinophils (%)	0.2
Basophils (%)	0.4
ADA	64.4
Acid-fast stain	No acid-fast bacilli seen
Gram stain	No bacteria seen

**Figure 2:**
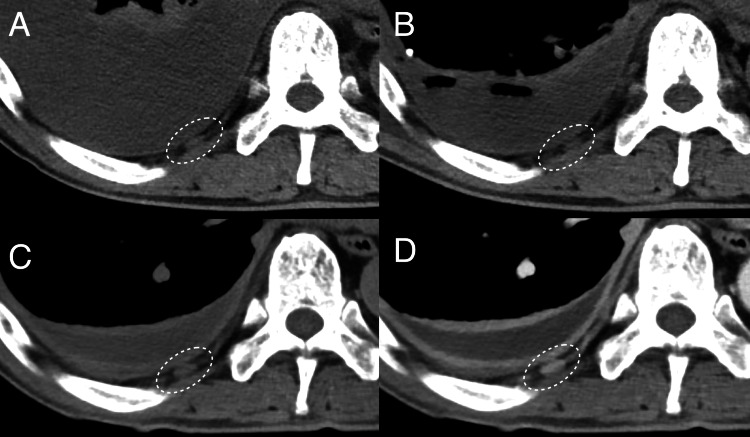
Close-up views of CT scans corresponding to Figures 1A-1D. (A, B) Close-up views before anti-tuberculosis therapy showing mild intercostal lymphadenopathy at the right ninth rib (dotted circles). (C, D) Close-up views 27 days after the initiation of anti-tuberculosis therapy, showing enlargement of the right ninth intercostal lymph node (dotted circles).

**Figure 3:**
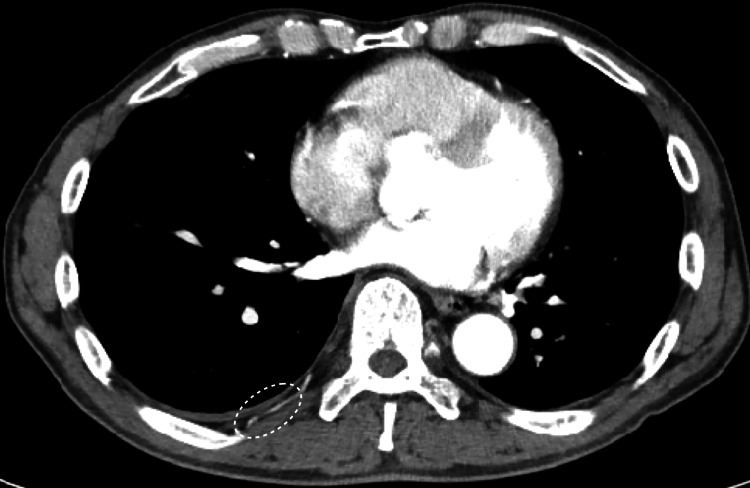
Follow-up CT imaging at nine months after discharge showing complete resolution of pleural effusion and intercostal lymphadenopathy (dotted circle).

On the 22nd day of anti-tuberculosis therapy, the patient developed right-sided back pain, recurrent fever (38.3°C), and a re-elevated CRP level (7.06 mg/dL). CT on the 27th day of anti-tuberculosis therapy showed no pulmonary infiltrates and an improvement of the pleural effusion (Figures 1C-1D). However, compared to the pre-treatment images, mild intercostal lymphadenopathy adjacent to the right ninth rib was observed (Figure 2), with corresponding tenderness over the right ninth rib. Magnetic resonance imaging on the 28th day of anti-tuberculosis therapy did not indicate an abscess or osteomyelitis of the right ninth rib. Based on physical examination and imaging studies, no other lymphadenopathy was observed, and there was no evidence of tumors or other infections. As the drug susceptibility testing showed susceptibility to all drugs, treatment failure or resistance was unlikely. Based on these findings, PR with intercostal lymphadenopathy due to anti-tuberculosis drugs was suspected. A biopsy was not performed due to the location of the lymph node. As the symptoms did not significantly affect the patient's quality of life, physical activity, or respiratory function, close monitoring was chosen without modifying treatment or adding corticosteroids, except for as-needed acetaminophen for pain. On the 34th day of anti-tuberculosis therapy, the right-sided back pain and fever resolved, and the CRP level improved (2.72 mg/dL). Based on this clinical course, the diagnosis of PR was established. The patient was discharged on day 65 of admission (58 days after the initiation of anti-tuberculosis therapy) and continued outpatient treatment. At the outpatient visit on the 113th day of anti-tuberculosis therapy, the CRP level had fully normalized (0.13 mg/dL). The anti-tuberculosis therapy was completed over six months, and during this period, there was no recurrence of tuberculosis or PR. Follow-up CT imaging at nine months after discharge demonstrated complete resolution of pleural effusion and intercostal lymphadenopathy (Figure 3). Two years have passed after the completion of treatment, and no recurrence of tuberculosis or PR has been observed.

## Discussion

This is a case of PR that developed during the treatment of tuberculous pleurisy, highlighting the importance of recognizing PR in tuberculosis treatment. Although the lymphadenopathy was mild, we believe that the localized pain in the right back is likely related to intercostal lymphadenopathy associated with the PR, based on the exclusion of other diseases and tenderness over the ninth rib directly above the lymph nodes.

PR is commonly recognized in the treatment of tuberculous cervical lymphadenitis and tuberculous central nervous system infections in non-HIV patients, as well as in patients with HIV and tuberculosis co-infection after the initiation of highly active antiretroviral therapy [1,2]. PR in tuberculous pleurisy was previously considered rare; however, a recent retrospective study found it to be relatively common, occurring in 16-27% of cases [2,3,8].

PR in tuberculous pleurisy often presents as increasing pleural effusion or the new appearance of pulmonary infiltration, with onset typically 7-8 weeks after the initiation of anti-tuberculosis therapy [2,3]. Previous case reports often described the onset of PR as worsening pleural effusion 1-2 months after treatment [9-11]. Retrospective reviews have suggested various risk factors for the development of PR in tuberculous pleurisy, including a low proportion of lymphocytes, a high proportion of polymorphonuclear cells, a high proportion of eosinophils, and low protein concentrations in the pleural fluid, as well as younger age and high serum albumin levels [2,3]. This case is rare in the following aspects: PR occurred early in anti-tuberculosis therapy and presented as intercostal lymphadenopathy. Given that tuberculous pleurisy is the second most common form of tuberculosis, clinicians should remain vigilant for PR when managing this condition, as it is easily misdiagnosed as treatment failure, drug-resistant tuberculosis, or another infection [3,5].

The differential diagnosis is broad due to the varied presentations of PR. Deterioration of tuberculous pleurisy after initiating anti-tuberculosis therapy should raise suspicion of PR. Other differential diagnoses include treatment failure, drug side effects, and complications associated with tuberculous pleurisy, such as empyema [7]. Additionally, since intercostal lymph nodes are typically not visible on imaging, their detection suggests pathological enlargement and warrants consideration of metastasis of malignancy, as well as non-neoplastic processes such as sarcoidosis, infections, or autoimmune diseases like systemic lupus erythematosus [12]. If drug susceptibility testing confirms multidrug-resistant tuberculosis, treatment should be adjusted accordingly before considering PR [13]. In our case, the patient experienced localized back pain due to intercostal lymphadenopathy. Drug susceptibility testing showed sensitivity to all drugs, and detailed physical examination and imaging studies helped differentiate from other conditions causing localized back pain, such as osteomyelitis or a skin abscess related to treatment failure.

PR in tuberculous pleurisy is usually mild, transient, and self-limited; therefore, the mainstay management is close monitoring. However, in cases with severe symptoms or massive pleural effusion compromising respiratory function, additional treatments, such as corticosteroids, may be required [2,3]. In our case, considering the severity of the disease and its impact on the patient's overall well-being, corticosteroids were not administered. The prognosis for PR is generally good. However, some literature suggests that careful observation is needed in cases of PR associated with central nervous system tuberculosis, as immediate investigation and anti-inflammatory treatment may be necessary [14].

There is no established diagnostic or treatment approach for suspected lymphadenopathy due to PR in tuberculous pleurisy. Park et al. investigated suspected PR-related lymphadenopathy in non-HIV tuberculous patients after anti-tuberculosis therapy. They found that at least 90% improved spontaneously, suggesting that conservative monitoring is generally appropriate [15]. Therefore, they proposed a stepwise approach, initially recommending a four-week observation period before considering further evaluation. If lymphadenopathy does not improve, a biopsy with mycobacterial cultures and drug susceptibility testing should be performed to assess the possibility of multidrug-resistant tuberculosis or other conditions. Improvement during the four-week observation period or negative biopsy results would confirm the diagnosis of lymphadenopathy due to PR, and monitoring should continue until resolution unless there are cosmetic concerns or quality of life impairments [15]. Therefore, as demonstrated in this case, when lymphadenopathy due to PR is suspected, spontaneous improvement can be expected, and conservative management without unnecessary tests or invasive interventions is advisable.

## Conclusions

Tuberculous pleurisy rarely presents with intercostal lymphadenopathy due to PR during anti-tuberculosis therapy. Although PR typically resolves without treatment, they are often misdiagnosed as treatment failure, leading to unnecessary invasive tests or treatments. During treatment for tuberculous pleurisy, unexplained deterioration of pleural effusion, pulmonary infiltrates, or intercostal lymphadenopathy should raise suspicion of PR, with close monitoring recommended for up to four weeks. This case highlights the importance of clinician vigilance for PR in tuberculosis management and the value of close monitoring without invasive interventions. A case series or retrospective analysis is needed to clarify the frequency and clinical features of intercostal lymphadenopathy as PR in tuberculous pleurisy.
